# *TaAP2-15*, An AP2/ERF Transcription Factor, Is Positively Involved in Wheat Resistance to *Puccinia striiformis* f. sp. *tritici*

**DOI:** 10.3390/ijms22042080

**Published:** 2021-02-19

**Authors:** Mehari Desta Hawku, Farhan Goher, Md Ashraful Islam, Jia Guo, Fuxin He, Xingxuan Bai, Pu Yuan, Zhensheng Kang, Jun Guo

**Affiliations:** State Key Laboratory of Crop Stress Biology for Arid Areas, College of Plant Protection, Northwest A&F University, Yangling 712100, China; hawku621@nwafu.edu.cn (M.D.H.); goherfarhan@nwafu.edu.cn (F.G.); a.islam160@nwafu.edu.cn (M.A.I.); guojia1889@nwafu.edu.cn (J.G.); hefx@nwafu.edu.cn (F.H.); baixingxuan@nwafu.edu.cn (X.B.); yuanpu00@nwafu.edu.cn (P.Y.)

**Keywords:** wheat, *Puccinia striiformis* f. sp. *tritici*, transcription factor, TaAP2, resistance, VIGS, salicylic acid

## Abstract

AP2 transcription factors play a crucial role in plant development and reproductive growth, as well as response to biotic and abiotic stress. However, the role of *TaAP2-15*, in the interaction between wheat and the stripe fungus, *Puccinia striiformis* f. sp. *tritici* (*Pst*), remains elusive. In this study, we isolated *TaAP2-15* and characterized its function during the interaction. TaAP2-15 was localized in the nucleus of wheat and *N. benthamiana*. Silencing of *TaAP2-15* by barley stripe mosaic virus (BSMV)-mediated VIGS (virus-induced gene silencing) increased the susceptibility of wheat to *Pst* accompanied by enhanced growth of the pathogen (number of haustoria, haustorial mother cells and hyphal length). We confirmed by quantitative real-time PCR that the transcript levels of pathogenesis-related genes (*TaPR1* and *TaPR2*) were down-regulated, while reactive oxygen species (ROS)-scavenging genes (*TaCAT3* and *TaFSOD3D*) were induced accompanied by reduced accumulation of H_2_O_2_. Furthermore, we found that TaAP2-15 interacted with a zinc finger protein (TaRZFP34) that is a homolog of OsRZFP34 in rice. Together our findings demonstrate that *TaAP2-15* is positively involved in resistance of wheat to the stripe rust fungus and provides new insights into the roles of AP2 in the host-pathogen interaction.

## 1. Introduction

It is estimated that global wheat (*Triticum aestivum*) yields are decreased by 3% to more than 90% per year due to the obligate biotrophic pathogen *Puccinia striiformis* f. sp. *tritici* (*Pst*), which threats global food supply [1,2]. The most economical, effective, and environment-friendly strategy to control this disease is breeding wheat-resistant varieties. Therefore, a deep understanding of the molecular mechanism of wheat resistance to *Pst* will allow us to develop new strategies for durably controlling stripe rust [2]. Plants lack a vertebrate-like circulatory system and antibodies to defend themselves from pathogenic attacks; instead, various forms of defense mechanisms have evolved in plants, including pathogen-associated molecular pattern (PAMP)-induced immunity (PTI, previously known as basal resistance), effector-induced immunity (ETI, previously known to as R gene-mediated resistance), and systemic acquired resistance (SAR) [3,4]. PTI and ETI involve dramatic transcriptional reprogramming in the plant, including upregulation of defense genes encoding antimicrobial proteins and enzymes for biosynthesis of anti-microbial secondary metabolites, as well as genes encoding proteins associated with signaling hormones [5,6].

In signal transduction, transcription factors (TFs) work primarily to control gene expression and the interactions between different signaling pathways [7,8]. These TFs code for proteins that bind to the *cis*-acting element in the region of the gene promoter to influence the expression of downstream genes that generate stress responses in eukaryotes [9,10]. TFs have been examined in many plants, including *Arabidopsis*, wheat, tomato, soya, rice, maize, and barley [11,12,13]. They serve a number of roles in plant growth processes and stress responses, such as vegetative and reproductive growth, cell proliferation, responses to abiotic and biotic stress, and responses to plant hormones [14,15,16]. In the *Arabidopsis* genome, more than 1500 genes encode transcription factors [17]. The APETALA2/Ethylene-Responsive Factor (AP2/ERF) superfamily has generated a great deal of interest in these TFs. This superfamily is divided into four major subfamilies based on the number and similarity of AP2/ERF domains: AP2 (APETALA2), RAV (related to ABI3/VP1), DREB (dehydration-responsive element-binding protein), and ERF [16,18]. AP2/ERF is one of the most important families of TFs in plants that regulate diverse developmental and stress responsive pathways via different mechanisms such as transcriptional and post-transcriptional control [19,20,21,22,23,24]. Recently, sixty-two AP2 gene family members were identified in wheat [25] and some of them have been reported to be involved in growth and development, and abiotic stress responses. In *Arabidopsis*, an AP2 gene, *ADAP* (ARIA-interacting double AP2-domain protein) has been reported to play a significant role in abscisic acid (ABA) and drought responses [26]. He et al. (2019) reported that *CsAP2-09* contributes to citrus canker disease resistance caused by *Xanthomonas citri* sbusp. *citri* [27]. The AP2 transcription factor family includes many genes that encode proteins involved in controlling disease resistance pathways [28]. It is worth noting that certain AP2s act in both biotic and abiotic stress tolerance, due largely to their participation in various pathways of hormonal signaling, such as ethylene, jasmonic acid (JA) or salicylic acid (SA). A common defense signaling pathway contributes to the accumulation of reactive oxygen species (ROS) and rapid influx of ions, accompanied by accumulation of SA, pathogenesis-related (PR) gene expression and cell death related to the hypersensitive response (HR) [29,30]. ROS production is one of the earliest cellular responses following pathogen recognition and the enhanced communication of ROS contributes to programmed cell death (PCD) by interfering with metabolism and destroying organelles in plant cells under abiotic and biotic stress [31,32]. PTI and ETI appear to be mediated by an integrated signaling network. However, activated immune responses in ETI are more prolonged and robust than those in PTI [30]. At the end of the phosphorylation cascade, TFs are triggered or suppressed by protein kinases or phosphatases and bind directly to *cis*-elements in stress-responsive gene promoters and thus control their transcription [33]. TFs themselves, however, are controlled by other upstream components at the transcription level [34] and also subjected to different levels of post-transcription modifications, such as ubiquitination and sumoylation, thus establishing a complex regulatory network to amplify the expression of stress-responsive genes, which in turn decide the activation of physiological and metabolic responses [35,36].

In various cellular functions, zinc finger proteins play a crucial role, including transcriptional control, RNA binding, apoptosis regulation, and protein-protein interactions [37]. Recent studies have shown that zinc finger family proteins play key roles in various development pathways, as well as in resistance and stress response pathways in plants [38,39]. In rice, a ubiquitin E3 ligase OsRZFP34 was found to be accumulated specifically in rice leaves at germination and vegetative stages after heat stress and ABA treatment [38]. OsPUB67 interacts with OsRZFP34 and positively regulates drought stress [39]. In plants, ligases related to E3 ubiquitin are key components of the ubiquitination cascade that leads to the response to various biotic and abiotic stresses.

AP2s have been shown to play an important role in previous studies, but little research has been done to understand the functions of those *AP2* genes in the interaction between wheat and *Pst*. In this study we identified and functionally characterized one AP2 gene, *TaAP2-15*, and dissected its important roles in wheat resistance to *Pst*. Furthermore, TaAP2-15 was found to interact with a zinc finger protein RZFP34/CHYR1, an ortholog of *Arabidopsis* AT5G25560 and rice OsRZFP34 (Os01t0719100). Thus, we concluded that *TaAP2-15* positively regulate wheat resistance against the stripe rust fungus. Our results provide new perspectives that contribute to our understanding of the functions of *AP2* gene families in the interaction of wheat with *Pst*.

## 2. Results

### 2.1. Sequence and Promoter Analysis

**Figure 1:**
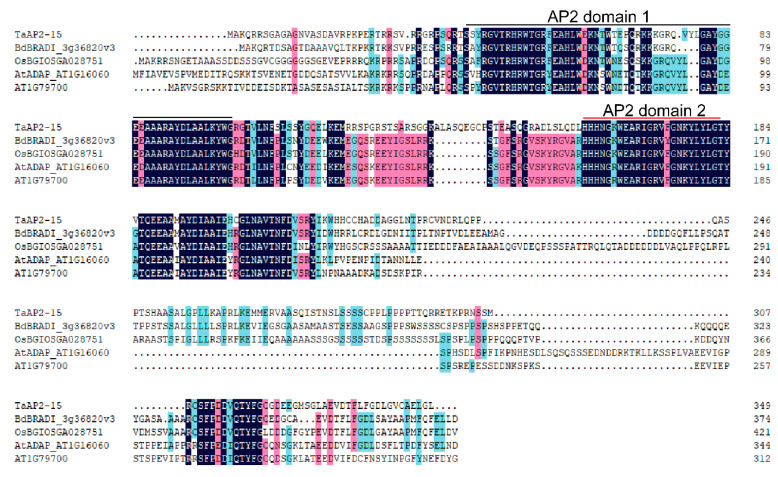
Multisequence alignment of TaAP2-15 with its orthologs in other plant species. Comparison of amino acid sequences of *Triticum aestivum* (*TaAP2-15*), with its orthologs from *Brachypodium distachyon* (BRADI_3g36820v3), *Oryza sativum* (BGIOSGA028751) and *Arabidopsis thaliana* (AT1G16060 and AT1G79700). Conserved residues through all organisms are shown in black (100%), pink (75–100%) and light blue (50–75%), respectively. Sequences alignment was performed using DNAMAN8.0 (Lynnon Biosoft, San Ramon, QC, Canada).

The full-length cDNA sequence of a previously designated *AP2* gene, *TaAP2-15* [19] was obtained from the cDNAs of wheat cv. Suwon 11 leaves infected with *Pst*. Blast analysis of *TaAP2-15* nucleotide sequence in the *Triticum aestivum* genome sequence revealed two copies, localized on chromosome 6A and 6B (Figure S1). The predicted open reading frame (ORF) of *TaAP2-15* contains 349 deduced amino acids with a molecular weight of 38.39 kDa and an isoelectric point (pI) of 9.47. Multisequence alignment indicated that TaAP2-15 is highly conserved with its orthologs from *Brachypodium distachyon* (BdBRADI_3g36820v3), *Oryza sativum* (OsBGIOSGA028751) and *Arabidopsis thaliana* (AtADAP_ AT1G16060 and AtAT1G79700) (Figure 1). Domain feature analysis indicated that TaAP2-15 contains two AP2 domains: AP2 domain 1 (amino acids 40-101) and AP2 domain 2 (amino acids 159–203) (Figure 1). Sequence analysis indicated that AP2 domain 1 of TaAP2-15 has high similarity with that of *Brachypodium distachyon* (87.3%), *Oryza sativum* (76.1%) and *Arabidopsis thaliana* (69.0% and 71.8%). In addition, AP2 domain 2 of TaAP2-15 shows high similarity with that of *Brachypodium distachyon* (94.6%), *Oryza sativum* (89.3%), and *Arabidopsis thaliana* (89.3% and 91.1%).

To gain further insight, an in silico analysis of possible *cis*-elements was performed. *Cis*-elements such as plant hormone-responsive motifs (TGACG-motif, P-box, CGTCA-motif) and low temperature stress responsiveness (LTR) were found within the promoter region of *TaAP2-15*. Interestingly, salicylic acid responsiveness (TCA-element) was also found in the promoter of *TaAP2-15* (Table 1).

### 2.2. TaAP2-15 Is Significantly Induced When Challenged with Pst

**Figure 2:**
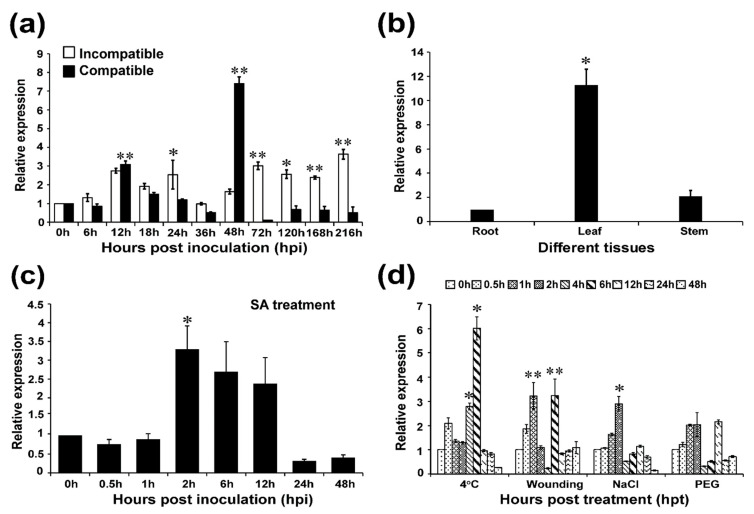
*TaAP2-15* is induced by multiple stresses. (**a**) *TaAP2-15* is induced in wheat leaves upon infection with *Pst* isolates, CYR23 (incompatible) and CYR31 (compatible) at diverse time points; (**b**) Expression of *TaAP2-15* in three wheat organs (root, leaf and stem), (**c**) Exogenous application of 2 nM SA in wheat leaves induced the expression of *TaAP2-15*, (**d**) The expression of *TaAP2-15* in wheat leaves is enhanced when treated with environmental stress (low temperature, wounding, salt and drought). Two-weeks-old wheat seedlings were used in all experiments. TaEF-1α was used as internal reference. Samples taken at each time point (uninoculated and untreated plants) were used as controls. Relative quantity of expression of *TaAP2-15* was computed via the comparative threshold (2^−ΔΔ*C*t^) method. The transcript levels were quantified by qRT-PCR and the values were standardized to those for *TaEF-1α* and presented as relative changes to untreated plants. The expression level of *TaAP2-15* at time 0 h was normalized as 1. Statistical variations were analyzed using Student’s *t*-test. *, *p* < 0.05, **, *p* < 0.01. All data were obtained from three biological replicas.

To confirm whether *TaAP2-15* participates in responses to *Pst*, transcript levels were determined by qRT-PCR. During the compatible interaction, the transcript profiles of *TaAP2-15* were up-regulated more than 7-fold at 48 h post inoculation (hpi) relative to control (Figure 2a). During the incompatible interaction, the transcript level of *TaAP2-15* was significantly induced at 12, 24, 72, 120, 168 hpi and reached a peak (more than 3.5-fold over that of the control) at 216 hpi. Therefore, we speculate that *TaAP2-15* participates in resistance of wheat to *Pst* as the transcripts during the incompatible interaction remain up-regulated in most of the time points compared to controls.

### 2.3. Tissue-Specific Expression of TaAP2-15

AP2/ERF family genes are expressed in various organs of the plant [25]. We examined the expression profiles of *TaAP2-15* in three different tissues (root, stem and leaf) of wheat plants. The results showed that the transcript levels of *TaAP2-15* were most abundant in leaf tissue, as high as 10-fold over the level in roots (Figure 2b).

### 2.4. TaAP2-15 Is Induced under SA Treatment and Abiotic Stress

Transcript levels of *TaAP2-15* in response to salicylic acid (SA), and abiotic stress were investigated via qRT-PCR. The transcript of *TaAP2-15* in response to salicylic acid (SA), was induced more than 3-fold as early as 2 h post treatment (hpt) and subsequently decreased at 6 and 12 hpt but still remained up-regulated compared to the control (Figure 2c). Various treatments were applied to investigate the role of *TaAP2-15* in response to abiotic stresses. As illustrated in Figure 2d, the transcripts of *TaAP2-15* were significantly induced in all except drought treatments compared to the control. After incubation at low temperature, the transcript profiles of *TaAP2-15* were up-regulated 6-fold over the control at 6 hpt. In response to wounding, the *TaAP2-15* transcripts were induced 3-fold higher than the control at 1 and 6 hpt. Salt treatment also significantly increased the transcript levels of *TaAP2-15* as high as 3-fold over the control at 2 hpt (Figure 2d). Taking these results together, we conclude that *TaAP2-15* is induced in the response of wheat to multiple stresses.

### 2.5. TaAP2-15 Is a Nuclear Localized Protein

**Figure 3:**
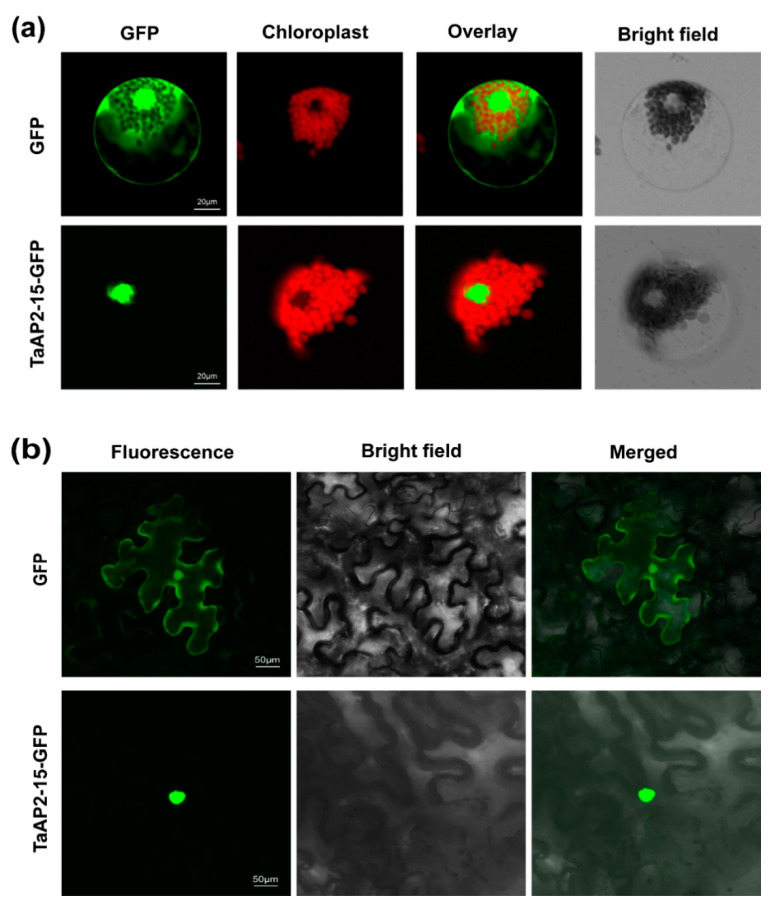
*TaAP2-15* encodes a nuclear targeted protein. (**a**) TaAP2-15-GFP fused proteins were overexpressed in wheat protoplasts through PEG-mediated transfection method. GFP was used as a control. (**b**) TaAP2-15-GFP fused proteins were overexpressed in tobacco through agrobacterium-mediated transformation. The constructs, GFP or TaAP2-15-GFP, were first introduced into *A. tumefaciens* then agroinfiltrated into *N. benthamiana* leaves. GFP signals were observed with an Olympus FV1000 confocal microscope with 488 nm filter.

To determine the subcellular localization of *TaAP2-15*, the generated constructs, p16318hGFP:*TaAP2-15* and pCAMBIA1302:*TaAP2-15*, were transiently expressed in wheat protoplasts and *N. benthamiana*, respectively. The empty vector, p16318hGFP, was used as a control. GFP was ubiquitously distributed throughout the cell, including in the nucleus (Figure 3a,b). The cells expressing GFP tagged with *TaAP2-15* were targeted in the nucleus of both wheat and *N. benthamiana* (Figure 3a,b). These results demonstrated that the *TaAP2-15* was expressed in the nucleus.

### 2.6. Silencing of TaAP2-15 Significantly Impaired Wheat Resistance to Pst

**Figure 4:**
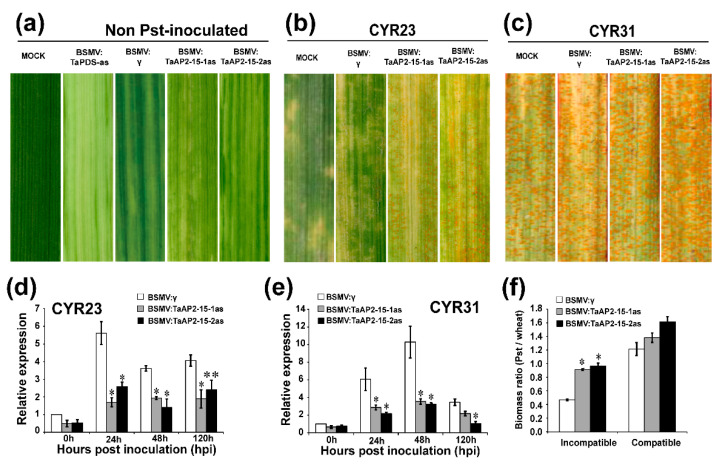
Silencing of *TaAP2-15* enhances wheat susceptibility to *Pst*. (**a**) Mild chlorotic mosaic virus symptoms on virus-inoculated plants. Mock: 1 x Fes buffer-treated leaves. (**b,c**) The reaction (infection type) of plants challenged with CYR23 (**b**) or CYR31(**c**) photographed 14 dpi. (**d,e**) Transcripts of *TaAP2-15* in silenced or control plants (plants inoculated with BSMV:γ) challenged with the avirulent CYR23 (**d**) and the virulent CYR31 (**e**). Relative quantity of the expression of *TaAP2-15* was computed by the comparative threshold (2^−ΔΔ*C*t^) method. The transcript levels of this gene were quantified by qRT-PCR. The data were normalized with the transcripts of the reference gene, *TaEF-1α* and are expressed as fold changes relative to the control (BSMV:γ) at 0 h. Data obtained from control plants (BSMV:γ) after *Pst* inoculation at 0 h were normalized as 1. (**f**) Fungal and wheat biomass ratio quantified from total genomic DNA content at 14 dpi. *PstEF* and *TaEF-1α* were used as internal references. Significant differences between *TaAP2-15*- knockdown and control plants computed using Student’s *t*-test are indicated by asterisks. *, *p* < 0.05, **, *p* < 0.01. All data were obtained from three biological replicas.

The functional role of *TaAP2-15* during wheat-*Pst* interaction was analyzed by BSMV-mediated-VIGS (virus induced gene silencing) as employed previously [40]. Two fragments within the coding region that are specific to *TaAP2-15* were designed for silencing purposes (Figure S1) and amplified with specific primers (Table S1). Two-weeks-old wheat seedlings were inoculated at the two-leaf stage with BSMV:*TaPDS*-as, BSMV:*TaAP2-15*-1as, BSMV:*TaAP2-15*-2as and BSMV: γ. Leaves inoculated with *TaPDS* showed strong photobleaching symptoms, whereas only mild symptoms of chlorotic mosaic were observed on BSMV:γ, TaAP2-15-1as and TaAP2-15-2as inoculated wheat leaves (Figure 4a), confirming that gene silencing was performed correctly. The fourth leaf of wheat plants that were pre-inoculated with BSMV:γ, TaAP2-15-1/2as were then inoculated with avirulent CYR23 or virulent CYR31 *Pst* isolates at 10 days post inoculation. At 14 days after inoculation with *Pst* CYR23, hypersensitive response (HR) symptoms were clearly observed on all leaves, mock or pre-inoculated with virus. Fungal uredia near the necrotic area were produced on leaves of *TaAP2-15*-silenced plants (Figure 4b). On the other hand, normal disease development was megascopically observed on CYR31 inoculated plants with slightly more spores on the silenced plants than the mock or BSMV:γ-inoculated plants (Figure 4c). To clarify whether *TaAP2-15* was silenced successfully, transcript levels of *TaAP2-15* were analyzed via qRT-PCR. The transcript levels in *TaAP2-15*-knockdown plants both in incompatible and compatible interactions were significantly reduced compared to control plants, indicating that *TaAP2-15* was successfully silenced (Figure 4d,e). The fungal biomass was also significantly increased in silenced plants during incompatible interactions (Figure 4f). These results suggested that *TaAP2-15* plays a positive role in wheat defense against *Pst*.

### 2.7. The Transcription of PR and ROS-Related Genes Is Influenced after Silencing of TaAP2-15

**Figure 5:**
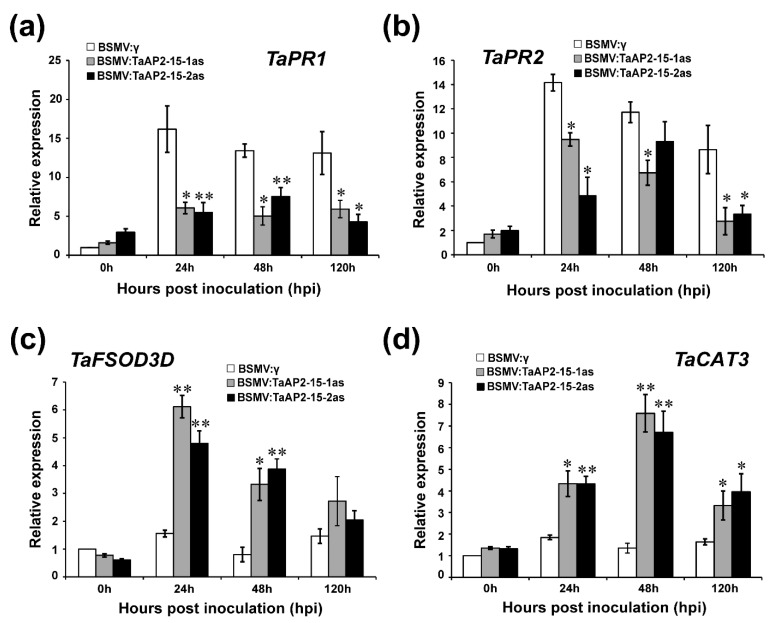
Relative expression of pathogenesis related (PR) and ROS-scavenging genes in *TaAP2-15*- silenced and control plants challenged with avirulent *Pst* race CYR23. The relative transcript levels of: (**a**) *TaPR1*, (**b**) *TaPR2* (*β*-1,3-glucanase) (**c**) *TaFSOD3D* and; (**d**) *TaCAT3* (catalase) were computed by qRT-PCR. Relative expression of these genes was computed by the comparative threshold (2^−ΔΔ*C*t^) method. The transcript levels of these genes were quantified by qRT-PCR and the data were normalized with the transcripts of the reference gene, *TaEF-1α* and expressed as fold changes relative to the control (BSMV:γ) at 0 h. Data obtained from control plants (BSMV:γ) after *Pst* inoculation at 0 h were normalized as 1. Significant differences between *TaAP2-15*-knockdown and control plants determined by Student’s *t*-test are indicated by asterisks. *, *p* < 0.05, **, *p* < 0.01. All data were obtained from three biological replicas.

To investigate whether silencing of *TaAP2-15* affects the expression of pathogenesis-related genes and ROS-scavenging genes, transcript levels of two PR-genes (*TaPR1* and *TaPR2*) and two ROS-scavenging genes (*TaCAT*3, catalase, and *TaFSOD3D*, iron superoxide dismutase) were examined by qRT-PCR. The expression patterns of both PR-genes (*TaPR1* and *TaPR2*) in the *TaAP2-15*-silenced plants were significantly down-regulated as compared to the control in both incompatible and compatible interactions (Figure 5a,b and Figure S2a,b). The transcripts of the ROS-scavenging genes, on the other hand, were significantly increased in *TaAP2-15*-silenced plants as compared to control plants (Figure 5c,d and Figure S2c,d). These results demonstrated that *TaAP2-15* positively regulates the expression of certain defense-related genes during the wheat-*Pst* interaction.

### 2.8. Silencing of TaAP2-15 Enhances Pst Growth and Decreases H_2_O_2_ Accumulation

**Figure 6:**
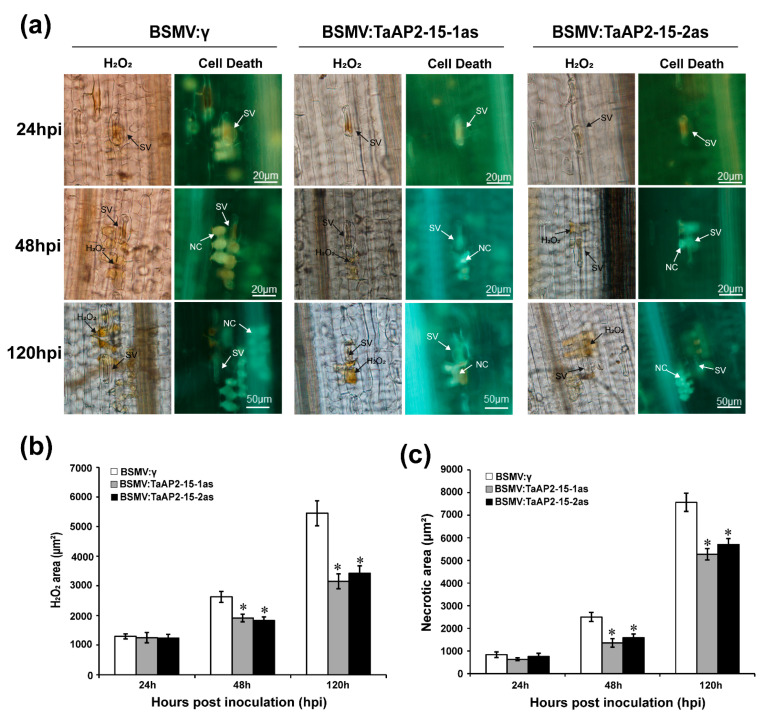
Knockdown of *TaAP2-15* enhanced wheat susceptibility to avirulent *Pst* race CYR23 infection. (**a**) Histological observations of wheat leaves treated with BSMV and infected with CYR23. Wheat leaves pre-infected with BSMV:γ, TaAP2-15-1/2as were subsequently inoculated with *Pst* race CYR23, and H_2_O_2_ and cell death were quantified. For H_2_O_2_ burst and detection of necrosis, *Pst* CYR23 inoculated leaves were sampled at 24, 48 and 120 hpi. These samples were then stained with DAB (3,3-diaminobenzidine). Microscopic examination (Olympus BX-51) was performed to assess H_2_O_2_ accumulation and necrosis around the infection sites. SV, substomatal vesicle; NC, necrotic cell. (**b**) H_2_O_2_ accumulation was quantified via DP-BSW software (Olympus, Tokyo, Japan) by measuring the area where DAB is visible at the infection site. (**c**) The area of cell death was determined by calculating the fluorescence area. All data are the means of ±SE of samples obtained from three independent biological replications. Data were computed from three biological replications and 50 infection sites. Significant differences between *TaAP2-15*-knockdown and control plants were estimated using Student’s *t*-test and are indicated by asterisks. *, *p* < 0.05.

**Figure 7:**
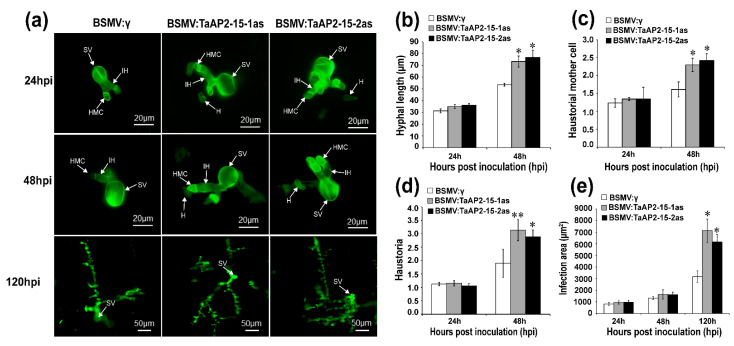
Silencing of *TaAP2-15* enhances growth of *Pst*. (**a**) Fungal structures in knocked-down and control plants challenged with *Pst* isolate CYR23. Leaves inoculated with *Pst* were sampled at 24, 48 and 120 hpi. Samples were then stained with WGA (wheat germ agglutinin) for fungal growth detection. Microscopy detection of different structures of *Pst* was performed by using Olympus BX-51 microscope. SV, sub-stomatal vesicle; IH, infection hypha; HMC, haustorial mother cells; H, haustoria. (**b**) Hyphal length as measured from the juncture of the hypha and substomatal vesicle to the tip of the hypha. DP-BSW tool (Olympus) was used to compute the hyphal length. (**c**) The average number of haustoria mother cells per individual infection site. (**d**) The average number of haustoria per individual infection site. (**e**) The colony area per individual infection point. Data were computed from three biological replications and 50 infection sites. Significant differences between *TaAP2-15*-knockdown and control plants were estimated using Student’s *t*-test and indicated by asterisks. *, *p* < 0.05, **, *p* < 0.01.

On the basis of phenotypic variation between control (BSMV:γ) and BSMV: TaAP2-15-1/2as after *Pst* inoculation, we evaluated the host responses and the fungal development. Accumulation of H_2_O_2_, which is strongly correlated to host resistance response, was measured and quantified via DP-BSW software in each infection site. Production of H_2_O_2_ in *TaAP2-15*-silenced wheat plants showed a substantial decrease in comparison to BSMV:γ-treated plants after *Pst* inoculation during the incompatible interaction (Figure 6a,b). Similarly, H_2_O_2_ accumulation was significantly decreased in *TaAP2-15*-silenced plants at 48 and 120 hpi during the compatible interaction (Figure S3a,b). The necrotic area in the *TaAP2-15*-knockdown plants was significantly lower than that of control plants at 48 and 120 hpi during the incompatible wheat-*Pst* interaction (Figure 6c). Microscopic examination indicated that the number of haustoria and haustoria mother cells and hyphal length were significantly increased in the *TaAP2-15*-silenced plants relative to the control at 48 hpi during both compatible and incompatible interactions (Figure 7a–d and Figure S4a–d). The infected area in both incompatible (Figure 7a,e) and compatible (Figure S4a,e) wheat-*Pst* interactions was also significantly increased in *TaAP2-15*-silenced plants at 120 hpi. Therefore, silencing of *TaAP2-15* enhances *Pst* growth and impairs plant defense to *Pst*.

### 2.9. TaAP2-15 Physically Interacts with TaRZFP34

**Figure 8:**
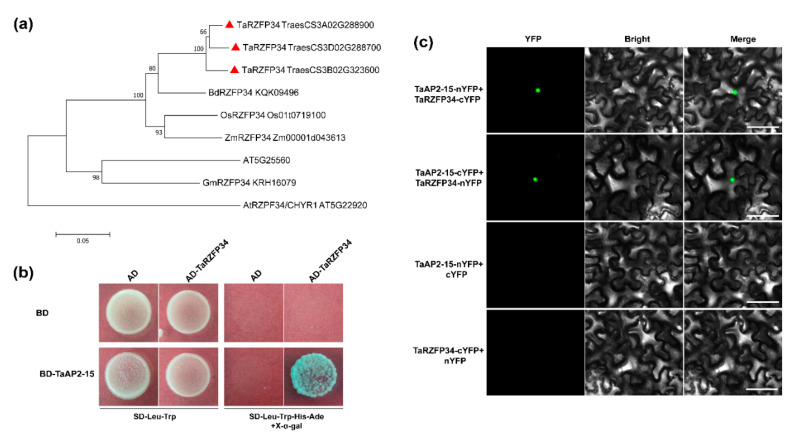
TaAP2-15 interacts with TaRZFP34. (**a**) Neighbor joining phylogenetic tree of TaRZFP34 with its orthologs. Bootstrap values shown at each node were estimated based on 1000 replications. Gene names and GenBank accession numbers are indicated. Ta, *Triticum aestivum*; Bd, *Brachypodium distachyon*; Os, *Oryza sativa*; *Zm*, *Zea mays*; Gm, *Glycine max*; At, *Arabdopsis thaliana*. The red triangle indicates three RZFP34 homologs in wheat. (**b**) TaAP2-15 interacts with TaRZFP34 verified by yeast two-hybrid. Constructs (pBD-TaAP2-15 and pAD-TaRZFP34) were plated on selective media SD-W/-L or SDW/-L/-H/-A (SD/-Trp/-Leu or SD/-Trp/- Leu/-His/-Ade containing 20 μg /mL X-α-gal). SD, synthetic dropout growth medium. (**c**) TaAP2-15 interacts with TaRZFP34 *in planta*. The constructs, TaAP2-15-nYFP+TaRZFP34-cYFP; TaAP2-15-cYFP+TaRZFP34-nYFP; TaAP2-15-nYFP+cYFP and TaRZFP34-cYFP+nYFP, were agroinfiltrated in to *N. benthamiana* leaves. After two or three days, samples from the infiltrated leaves were examined for fluorescent signals under a microscope. Bar, 100 μm.

To identify the target protein, yeast-two hybrid (Y2H) screening was carried out from a cDNA library constructed from *Pst* infected wheat cv. Su11. *TaAP2-15* was used as a bait for screening the cDNA library. A wheat zinc-finger protein, TaRZFP34 homolog of *Arabidopsis* AT5G25560 and rice OsRZFP34 (Os01t0719100) which encodes RING E3 ligase containing 299 amino acids, was identified (Figure 8a and Figure S5a,b, Table S2). To verify the interaction, the ORF sequence of *TaAP2-15* was subcloned into the bait vector (pBD-TaAP2-15), whereas the *TaRZFP34* was inserted into the prey vector (pAD-TaRZFP34) and co-expressed and grown in a selective medium lacking leucine, tryptophan, histidine and adenine but containing X-α-Gal. The interaction between TaAP2-15 and TaRZFP34 was confirmed by growth of yeast strain AH109 on selective medium staining with X-a-gal (Figure 8b). In vivo interaction between TaAP2-15 and TaRZFP34 was further verified by biomolecular fluorescence complementation (BiFC) assay. The following constructs including TaAP2-15-nYFP+TaRZFP34-cYFP, TaAP2-15-cYFP+TaRZFP34-nYFP, TaAP2-15-nYFP+cYFP and TaRZFP34-cYFP+nYFP, were generated for BiFC assay. The constructs were then transformed into *A. tumefaciens*, strain GV3101 and co-infiltrated into *N. benthamiana* leaves. Microscopic examination detected fluorescence only when the two constructs were co-expressed. No fluorescence was observed in leaves agroinfiltrated with either of the constructs mixed with the control (empty vector) (Figure 8c).

## 3. Discussion

The conserved AP2 DNA binding domains of amino acids 57-66 characterize the AP2/ERF superfamily [41]. In accordance with the double AP2 domain amino acid sequence and the nuclear location sequence, the AP2 family was subdivided into euAP2, eu-AINTEGUMENTA (euANT) and basalANT groups, respectively [42,43,44]. AP2 TFs play a number of roles in plant developmental processes and stress responses, such as vegetative and reproductive growth, cell proliferation, abiotic and biotic stress responses, and plant hormone responses [14,15,16]. ERFs often become up-regulated in hormonal and stress responses [45,46], and usually, these responses are immediate [27]. In order to understand plant adaptation to biotic stresses, research into the functions and mechanisms of wheat AP2/ERFs in the regulation of biotic stress responses is vital. Recent findings have shown that AP2 proteins perform a significant role in controlling responses to biotic and abiotic stress in plants.

In this study, qRT-PCR assays revealed that during the compatible interaction the transcript profiles of *TaAP2-15* were highly up-regulated at one time point (48 hpi). However, during the incompatible interaction, the transcript levels were significantly induced at all the time points, and even reached to its peak at 216 hpi. During the incompatible interaction, induction of the *TaAP2-15* transcript was generated faster and to a greater extent than those in the compatible interaction. In response to stress, gene expression triggered by environmental stresses not only protects cells from injury, but also controls the expression of genes involved in signal transduction processes [8]. Several studies have indicated that expression of ERFs was induced and up-regulated by abiotic stresses [47,48]. We found that expression of *TaAP2-15* was significantly induced by abiotic stresses (cold, wound, salt and drought), implying that *TaAP2-15* is induced in defense responses of wheat to multiple abiotic stresses. It was previously reported that AP2/ERF family members are expressed in various parts of the plant [25]. *TaAP2-15* was most abundantly expressed in wheat leaves. Like other TFs, AP2s have also been reported to be localized in nucleus. He et al. (2019) reported that *CsAP2-09* from citrus was localized to the nucleus of onion cells [27]. Similarly, in our study, *TaAP2-15* was found to be localized within the nuclei of both wheat and *N. benthamina*.

Our current research shows that *TaAP2-15* is required for resistance of wheat to infection by *Pst*. As a biotrophic parasite, *Pst* is dependent on haustoria to consume nutrients from the host [49]. By 48 hpi, the primary infection develops hypha, haustorial mother cell, and haustorium in both compatible and incompatible interactions, whereas the necrotic area in *TaAP2-15-*silenced plants was lower than the control at 48 and 120 hpi during the incompatible interaction. HR, or necrotic cell death, is the host defense reaction in response to invading pathogens. So, when resistant-related genes are silenced, it is obvious that necrotic cell death would be reduced in the silenced plants. In our findings, the reduced necrotic area in *TaAP2-15-*silenced plants indicated that *TaAP2-15* contributes to resistance. Furthermore, the fungal biomass was also increased in *TaAP2-15-*silenced plants during the incompatible interaction. It has been documented that, during pathogen infection and environmental stress, the abiotic and biotic stresses that trigger an oxidative burst response act as a defense system [50,51].

One of the earliest signaling events in plants, the ROS burst, occurs in the early stages of plant-pathogen interactions [52]. Previous histological and cytological findings in wheat have shown that in incompatible interactions with *Pst*, the activation of response signaling is associated with bursts of ROS as early as 12 hpi [53]. In our study, silencing of *TaAP2-15* decreased the content of H_2_O_2_ that is correlated with host resistance. The ROS burst is regulated by ROS-related genes. As demonstrated herein, the transcript levels of the ROS-related genes were significantly increased in *TaAP2-15*-silenced plants as compared to control plants. This finding agrees with earlier studies indicating that the induction of ROS accumulation in the signaling of biotic and abiotic stress is correlated with the ROS burst [54]. Transcription factors of ERF directly control the expression of pathogenesis-related (PR) genes [55,56,57]. The transcript levels of PR-genes, *TaPR1*, *TaPR2*, in the *TaAP2-15*-silenced plants were down-regulated compared to controls in both compatible and incompatible interactions, suggesting that *TaAP2-15* regulates the expression of *TaPR1* and *TaPR2* directly or indirectly. Phytohormones like SA have been reported to play significant roles in abiotic stress responses in plants [58]. SA is a basal and SAR signaling molecule with HR activation during biotrophic invasion. Vlot et al. (2009) reported that SA-mediated signaling pathway is activated upon infection with biotrophic pathogens [59]. Furthermore, SA was induced upon infection with *Pst* [60]. Some transcription factors like AP2/ERFs have been implicated indirectly in regulating the SA response, perhaps through interaction with other TFs [28]. Here, we showed that the transcript abundance of *TaAP2-15* was stimulated by application of exogenous SA. In *Arabidopsis*, accumulation of the plant hormone SA and transcriptional activation of PR genes is associated with SAR [61]. During host-pathogen interactions, PRs accumulate systemically [62], and in our study the accumulation of PR genes in silenced plants was diminished. Thus, we speculate that *TaAP2-15* is required positively to acquire resistance against *Pst* infection.

To summarize, our data indicate that *TaAP2-15* acts as positive regulator in resistance of wheat against *Pst* in a SA-induced pattern via ROS-mediated defense pathway. Analysis of the promoter region of *TaAP2-15* further revealed the presence of a SA responsive TCA-element, a potential *cis*-acting element for SA responsiveness. Our findings also imply that TaAP2-15 physically interacts with TaRZFP34, a zinc finger protein homolog in rice that has been reported to play a role in stress conditions. Here, we hypothesize that TaAP2-15 and its co-protein are activated through induction by SA treatment as the result of downstream signaling pathways that confer ROS-mediated defense. More studies are required to define these phenomena. To the best of our knowledge, this is the first study indicating that the wheat AP2 is positively required for resistance against stripe rust fungus infection.

## 4. Materials and Methods

### 4.1. Plant Material, Fungal Pathogens and Inoculation/Treatments

Wheat (*T. aestivum* L.) cultivar Suwon11 (Su11) and *Pst* isolates, CYR23/avirulent and CYR31/virulent, were used. Su11 possesses a *Pst* resistance gene *YrSu*. This cultivar is reported to be resistant to CYR23 but susceptible to CYR31 [63]. For RNA isolation, wheat leaves challenged with these *Pst* isolates or water (distilled and sterile) were sampled at different time points [53].

The expression of *TaAP2-15* in response to hormonal and environmental stress treatments was assessed. To investigate the transcripts of *TaAP2-15* to hormone treatment, 2 nM salicylic acid (SA) was applied through spraying to leaves of wheat seedlings. Leaves subjected to SA or 0.1% (*v*/*v*) ethanol (mock) treatments were then sampled for RNA isolation. To verify the response to drought and salinity, roots of wheat seedlings were immersed in 200 mM NaCl or 20% PEG6000, respectively. To assess the reaction of *TaAP2-15* in response to low temperature, wheat seedlings were maintained at 4 °C for 48 h. Wheat seedlings were also subjected to wounding by removing the tip of each leaf. Samples were collected at 0, 0.5, 1, 2, 4, 6, 12, 24 and 48 hpt from SA and different stress elicitors (NaCl, PEG 6000, low temperature and wound) treated seedlings. To determine tissues-specific expression of *TaAP2-15*, qRT-PCR analysis was carried out on different plant organs of two-weeks-old wheat seedlings. The experiment was repeated three times.

### 4.2. RNA Extraction, cDNA Synthesis and qRT-PCR Analysis

RNA was isolated with the Trizol reagent according to the manufacturer’s instructions (Invitrogen, Carlsbad, CA, USA) and treated with DNase I (Progema, Madison, WI, USA) to remove DNA contamination. cDNA was then synthesized using GoScript Reverse Transcription System (Progema) and an oligo (dT18) primer (Invitrogen). Primers specific to the gene of interest or reference gene were used to quantify the expression level by qRT-PCR using the synthesized cDNA [64] (Table S1). A 7500 Real-Time PCR System (Applied Biosystems, Foster City, CA, USA) was used to quantify the transcripts. *TaEF-1α* (GenBank accession Q03033) was used as the internal reference for normalizing the data. The relative expression of *TaAP2-15* was determined by the comparative 2^−ΔΔ*C*t^ [65] method. All qRT-PCR experiments were carried out with three replications using RNA samples obtained from three independent replicates.

### 4.3. Identification, Sequence and Promoter Analysis of TaAP2-15

A 1050-bp nucleotide sequence (accession number TraesCS6A01G125700) with high homology to *A. thaliana* homolog, AT1G16060 ADAP, which is positively involved in hormone and stress response [26] was obtained from the cDNA library in our lab [54]. This gene was previously designated as *TaAP2-15* [25]. *TaAP2-15* was amplified from *Pst* inoculated cDNA template using specific primers (Table S1). The cDNA sequence of *TaAP2-15* was further analyzed with the NCBI BLAST program (http://www.ncbi.nlm.nih.gov/blast/ (accessed on 17 February 2021)) and the ORF finder software in NCBI. The conserved domain was predicted with Pfam (http://pfam.sanger.ac.uk/ (accessed on 7 February 2021)), PROSITEScan (http://prosite.expasy.org/scanprosite/ (accessed on 7 February 2021)) and InterproScan (http://www.ebi.ac.uk/Tools/pfa/iprscan/ (accessed on 7 February 2021)). Multiple sequence alignments were performed using DNAMAN8.0 (Lynnon Biosoft).

To investigate the possible regulatory mechanisms of *TaAP2-15* gene, a 1.5-kb promoter region upstream of the start codon of the gene was retrieved from the wheat Genome Database (http://plants.ensembl.org/ (accessed on 7 February 2021)) and analyzed through the PlantCARE (http://bioinformatics.psb.ugent.be/webtools/plantcare/html/ (accessed on 7 February 2021)), an online server.

### 4.4. Subcellular Localization of TaAP2-15 Protein

The subcellular localization of *TaAP2-15* was determined in both wheat and tobacco (*N. benthamiana*). The ORF of *TaAP2-15* was constructed into p16318hGFP or pCAMBIA1302 vectors. The constructs, p16318hGFP-*TaAP2* and p16318hGFP were then introduced into the isolated protoplasts of wheat mesophyll tissue by the PEG-mediated transformation method [66]. To further confirm the localization of the TaAP2-15 protein, pCAMBIA1302:TaAP2-15 fusion was transformed into *A. tumefaciens* strain GV3101 by electroporation and infiltrated into tobacco leaves as described [67]. In a dark chamber, the transformed wheat protoplasts were incubated for 24–36 h at 24 °C. Infiltrated *N. benthamiana* leaves were also maintained in a growth chamber with a 16 h/8 h photoperiod at 25 °C for 2 to 3 days. Leaf tissue samples were then collected for detection of the autofluorescence signals with an Olympus FV1000 confocal laser microscope with a 480-nm filter [68].

### 4.5. BSMV-Mediated TaAP2-15 Gene Silencing

Gene silencing was performed through the BSMV-VIGS-mediated gene silencing method [69]. To silence *TaAP2-15*, two cDNA fragments of *TaAP2-15* (Figure S1) were selected and constructed into BSMV-γ-vector, resulting in constructs BSMV-γ:TaAP2-15-1as/2as. Specificity of the fragments was confirmed through BLAST analysis (http://blast.ncbi.nlm.nih.gov/Blast/ (accessed on 7 February 2021)). Capped in vitro transcripts were obtained with the RiboMAXTM Large-Scale RNA Production System-T7 (Promega, Madison, WI, USA) and Ribom7G Cape Analog (Promega) based on the manufacturer’s protocol. Barley stripe mosaic virus constructs were inoculated onto the second leaf of wheat as described previously [40] and plants were maintained in darkness at 25 ± 2 °C for 24 h with sufficient humidity. Mock plants were inoculated with 1 × Fes. Ten days later, freshly collected urediniospores of *Pst* race CYR23 or CYR31 were inoculated onto the fourth leaf. Leaf samples inoculated with *Pst* were then collected at 0, 24, 48 and 120 hpi for histology and RNA extraction. Silencing efficiency of the *TaAP2-15-*silenced plants and relative expression of pathogenesis (PR) and ROS-related genes were quantified by qRT-PCR analysis compared to control plants (BSMV:γ). After 14 days, the level of infection of *Pst-*inoculated leaves was determined based on the McNeal measurement scale [70] and infection phenotypes were photographed. Genomic DNA was extracted by the CTAB method from *Pst-*inoculated leaves collected after 14 days. Fungal biomass was then quantified by qRT-PCR, and a standard curve was generated from the plasmid carrying the fragments of *PsEF1* and *TaEF1α* [71]. The experiment was performed with three biological replications.

### 4.6. Histology of Fungal Growth and Host Response

Wheat leaves inoculated with *Pst* isolates were sampled at 24, 48 and 120 hpi and stained to detect the accumulation of H_2_O_2_ and fungal structures. H_2_O_2_ production was examined by staining samples with 3,3′-diamino benzidine stain (DAB) as described previously [53] and viewed under BX-51 microscope (Olympus). Wheat germ agglutinin stain (WGA) (Invitrogen) was used to visualize pathogen structures. During the wheat-*Pst* interaction, the formation of a substomatal vesicle is considered to be effective penetration [72]. At least 50 infection regions were examined for each treatment to assess the H_2_O_2_ accumulation, necrotic area and various fungal structures. Necrotic cells around the infected site, H_2_O_2_ accumulation and fungal structures, such as hypha, haustoria mother cell and haustoria, were observed with BX-51 microscope (Olympus) and their corresponding lengths were estimated using DP-BSW software. Student’s *t*-test was used to compute the statistical differences between treatments.

### 4.7. Yeast Two-Hybrid Assay

MatchMaker yeast two-hybrid assay (Clontech, Tokyo, Japan) was carried out to analyze the interaction between TaAP2-15 and TaRZFP34. The full-length sequence of TaAP2-15 was subcloned into pGBKT7-BD to generate TaAP2-15-BD, the DNA-binding domain bait protein fusion. A cDNA library generated from virulent *Pst* isolate-infected wheat leaves in the pGADT7 vector was used as a prey for screening the target gene. The bait (TaAP2-15-BD) and the prey (pGADT7-cDNA libraries) were co-transformed into a yeast strain AH109 and grown in a selective medium (SD/-Trp-Leu or SD/-Trp-Leu-His). The colonies grown on the SD/-Trp-Leu-His were then re-plated on SD medium lacking -Trp-Leu-His-Ade to check the interaction. The positive colonies were isolated, sequenced and analyzed by blasting on the NCBI database for the coding cDNA sequence (Table S1). Those candidate genes were then subcloned in to pGADT7 to generate the activation domain, AD prey protein fusion. To verify the interaction, TaAP2-15-BD and each candidate target gene in the AD vector were co-transformed into yeast and plated on SD/-Trp-Leu-His-Ade medium containing X-α-gal.

### 4.8. BiFC Assays

The ORF sequence of *TaAP2-15* was cloned into a pUC-PSYNE vector and fused with the N-terminal fragment of the yellow fluorescent protein (YFP) to generate *TaAP2-15*-nYFP construct. The full-length coding sequence of *TaRZFP34* was subcloned into a pUC-pSPYCE vector as a fusion with the C-terminal fragment of YFP to generate *TaRZFP34*-cYFP [73]. To verify if there is change in expression with exchange of the constructs in the vectors, we cloned *TaAP2-15* into pUC-PSYCE to produce *TaAP2-15*-cYFP and *TaRZFP34* into pUC-PSYNE to form *TaRZFP34*-nYFP. All the generated constructs TaAP2-15-nYFP, TaRZFP34-cYFP, TaAP2-15-cYFP, TaRZFP34-nYFP; TaAP2-15-nYFP+cYFP and TaRZFP34-cYFP+nYFP were then introduced into *A. tumefaciens* strain GV3101. The agrobacterium colonies containing the appropriate constructs were then infiltrated into 4-week-old *N. benthamiana* leaf tissue. In vivo interaction was then detected under a FV3000 confocal laser microscope 48 h after infiltration (Olympus).

### 4.9. Statistical Analysis

All data were subjected to analysis with Microsoft Excel. The statistical differences between treatments were computed by Student’s *t*-test.

## Figures and Tables

**Table 1:** Promoter analysis of *TaAP2-15.*

Site Name	Position	Strand	Sequence	Function
TCA-element	147	−	CCATCTTTTT	Salicylic acid response
TGACG-motif	362	+	TGACG	MeJA response
P-box	1432	+	CCTTTTG	Gibberellin response
LTR	315	−	CCGAAA	Low temperature response
CGTCA-motif	362	−	CGTCA	MeJA response

## Data Availability

Data is contained within the article and supplementary material.

## Supplementary Materials

Supplementary materials are available at: https://www.mdpi.com/1422-0067/22/4/2080/s1, Figure S1: Multiple sequence alignment of the ORF sequences for the *TaAP2-15* copies, Figure S2: Relative expression of pathogenesis related (PR) and ROS-scavenging genes in *TaAP2-15* silenced and control plants challenged with virulent *Pst* race CYR31, Figure S3: Knockdown of *TaAP2-15* enhanced wheat susceptibility to virulent *Pst* race CYR31 infection, Figure S4: Silencing of *TaAP2-15* in wheat enhances growth of the *Pst* virulent race, CYR31, Figure S5: Multiple sequence alignment of *TaRZFP34*, Table S1: Primers used in this study, Table S2: Candidate interacting genes screened via Y2H.
