# Supplementary material for: TaAP2-15, An AP2/ERF Transcription Factor, Is Positively Involved in Wheat Resistance to Puccinia striiformis f. sp. tritici
Source: Int J Mol Sci. 2021 Feb 19;22(4):2080. doi: 10.3390/ijms22042080 (PMC7923241; doi:10.3390/ijms22042080)
Supplement: Supplementary file 1 [file ijms-22-02080-s001.pdf]

SUPPLEMENTARY DATA

Article title: *TaAP2-15*, an AP2/ERF transcription factor, is positively involved in Wheat Resistance to *Puccinia striiformis* f. sp. *tritici*

Authors: **Mehari Desta Hawku, Goher Farhan, Md Ashraful Islam, Jia Guo, Fuxin He, Xingxuan Bai, Pu Yuan, Zhensheng Kang and Jun Guo**

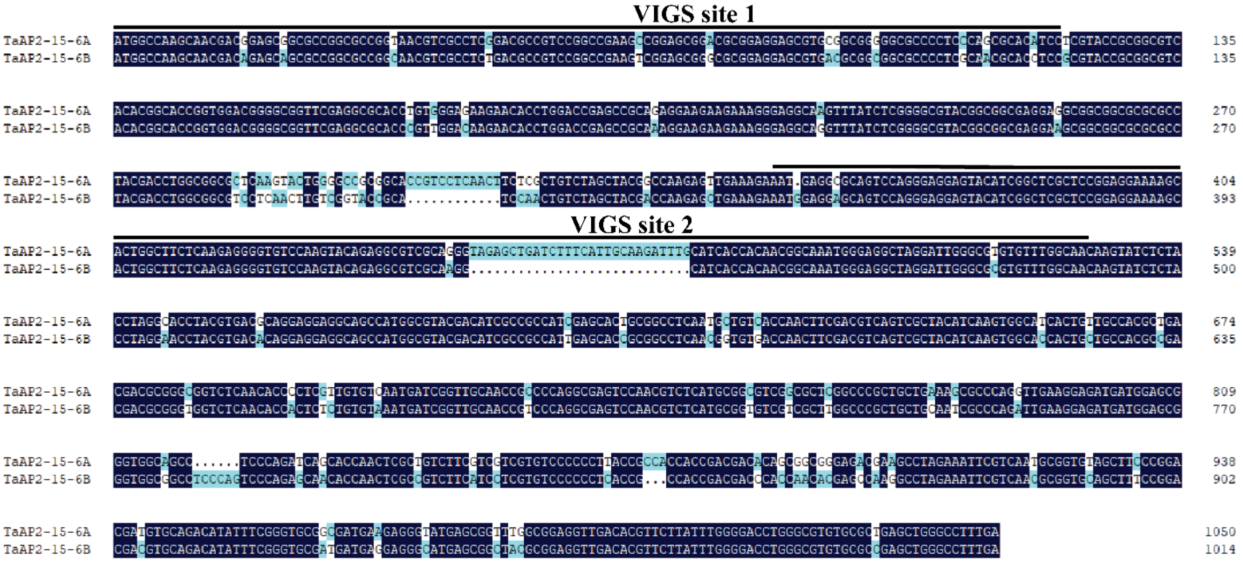

Figure S1. Multiple sequence alignment of the ORF sequences for the *TaAP2-15* copies**.** DNAMAN8 software (Lynnon Biosoft, USA) was used for alignments of the two *TaAP2-15* copies obtained from wheat cv. Chinese Spring genome database or cloned from wheat cv. Su11. The fragments for VIGS are indicated by overbars. VIGS site 1 and site 2 represent for *TaAP2-15* -1as and *TaAP2-15*-2as, respectively.

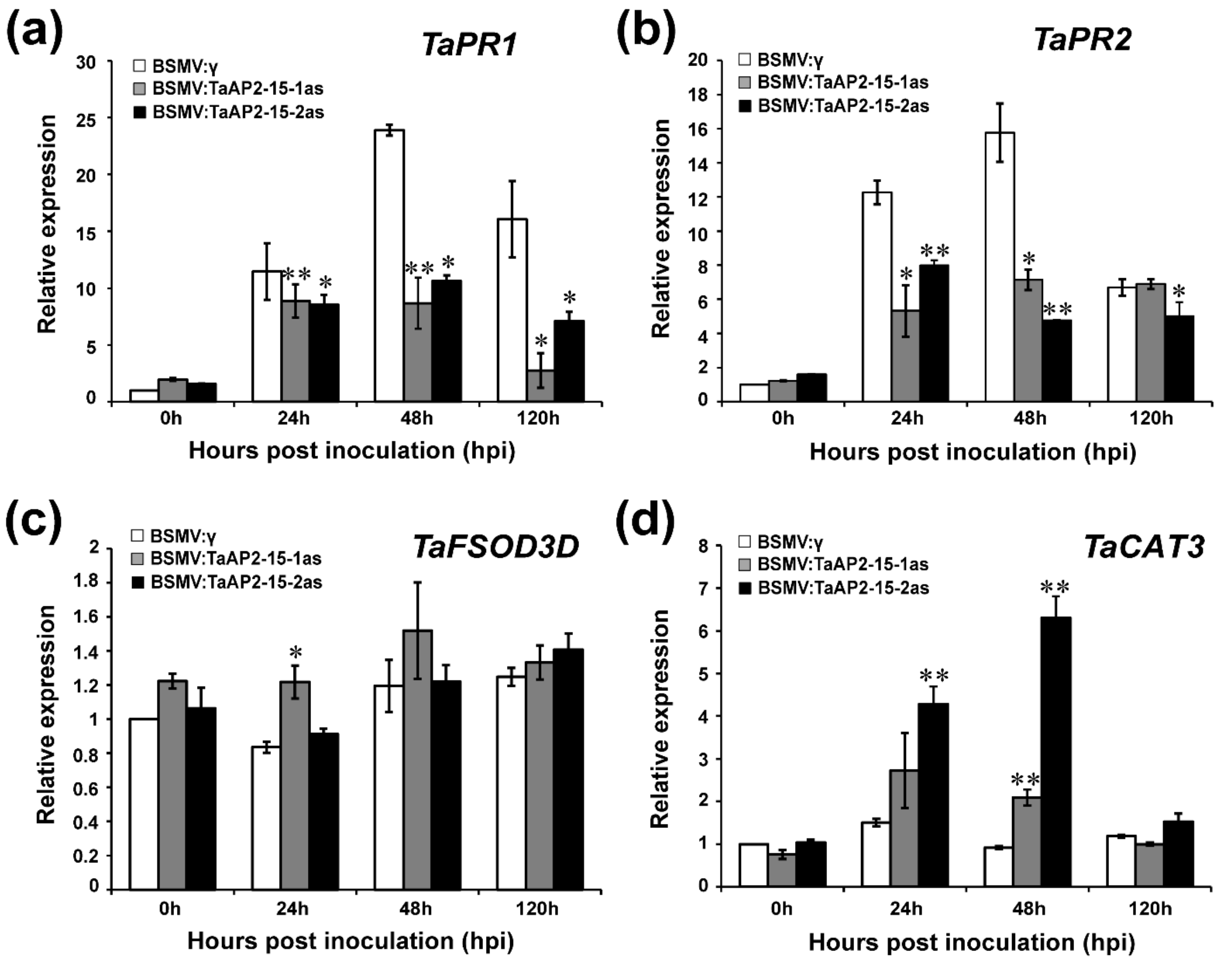

Figure S2. Relative expression of pathogenesis related (PR) and ROS-scavenging genes in *TaAP2-15*-silenced and control plants challenged with virulent *Pst* race CYR31. The relative transcripts of: (**a**) *TaPR1*, (**b**) *TaPR2* (β-1,3-glucanase) (**c**) *TaFSOD3D* and; (**d**) *TaCAT3* (catalase) was computed using qRT-PCR. Relative quantity of expression of these genes was computed using comparative threshold ($2^{-\Delta\Delta Ct}$) method. The transcript levels of these genes were quantified using qRT-PCR and the obtained data were normalized with the transcripts of the reference gene, *TaEF-1α* and demonstrated as fold changes relative to control (BSMV:γ) at 0h. Data obtained from control plants (BSMV:γ) after *Pst* inoculation at 0h was normalized as 1. The significant difference between *TaAP2-15*-knockdown and control plants is indicated by asterisks, which was computed with Student's *t*-test. *, $p < 0.05$, **, $p < 0.01$.

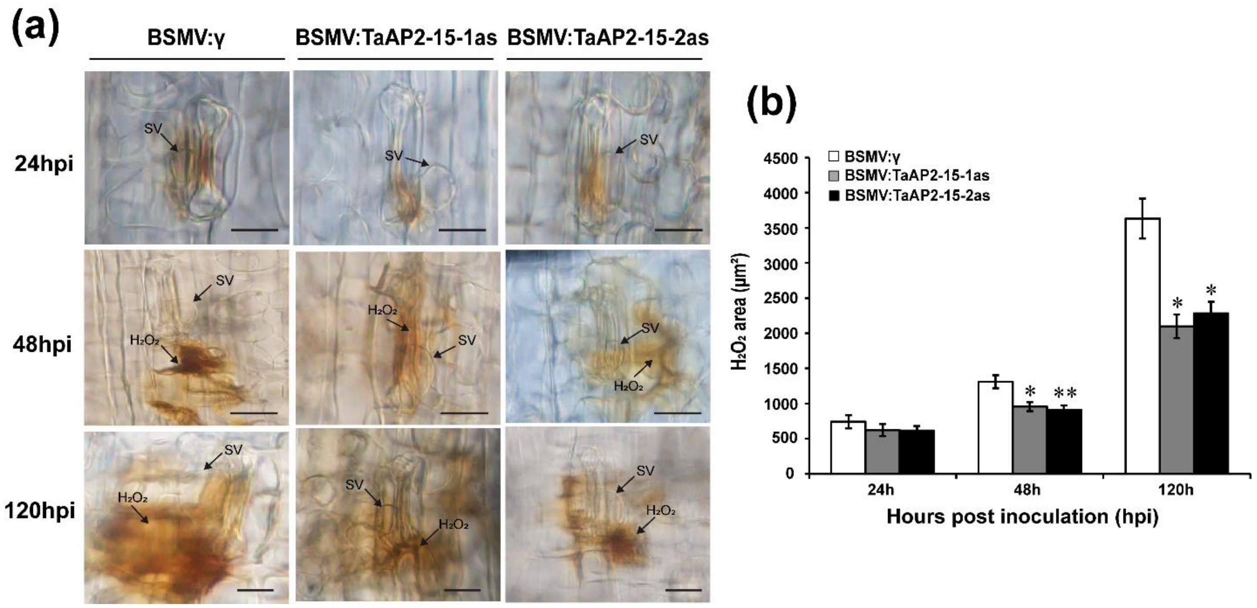

Figure S3. Knockdown of *TaAP2-15* enhances wheat susceptibility to virulent *Pst* race CYR31 infection. (**a**) Infection site and H_2O_2 accumulation was detected under BX-51 microscope (Olympus, Tokyo, Japan). Wheat leaves pre-infected with BSMV:γ, TaAP2-15-1/2as were subsequently inoculated with *Pst* CYR31, and H_2O_2 was quantified. For H_2O_2 burst detection, wheat leaves inoculated with *Pst* CYR31 were sampled at 24, 48 and 120 hpi. These samples were then stained with DAB (3,3-diaminobenzidine). Microscopy examination was performed to assess H_2O_2 accumulation. SV, substomatal vesicle; (**b**) H_2O_2 accumulation was quantified via DP-BSW software (Olympus, Tokyo, Japan) by measuring the area where DAB is stained at the infection site. Data are means of ± standard errors of three independent biological samples. Data were computed from three biological replications and 50 infection sites. The significant difference between *TaAP2-15*-knockdown and control plants is indicated by asterisks, which was estimated using Student's *t*-test. *, $p < 0.05$.

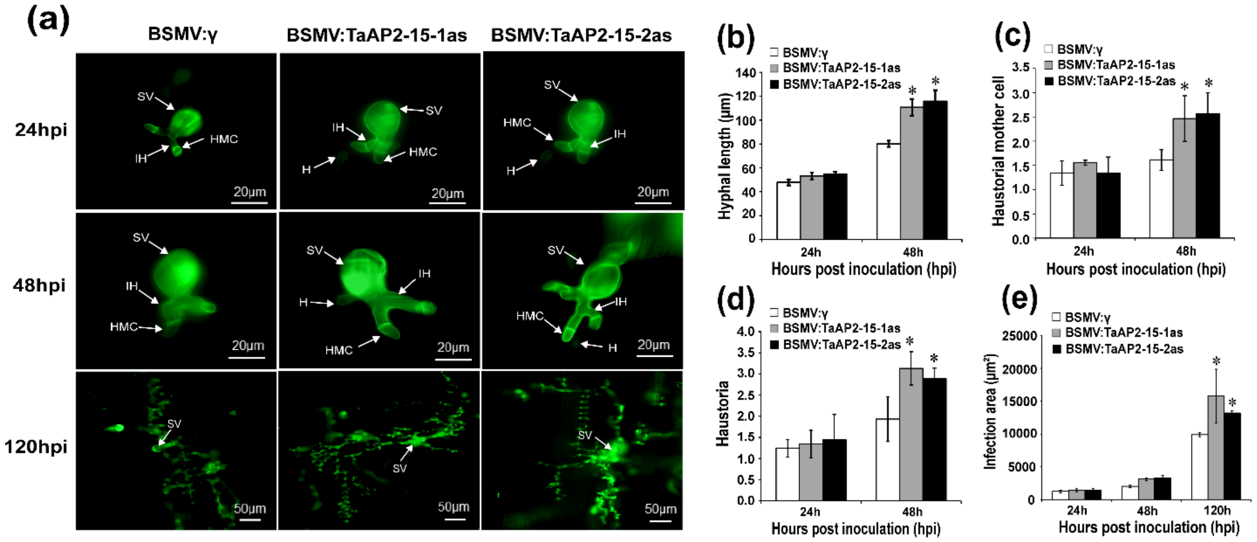

Figure S4. Silencing of *TaAP2-15* in wheat enhances growth of the *Pst* virulent race, CYR31. (**a**) Fungal structures in knocked-down and control plants inoculated with *Pst* isolate CYR31. *Pst* inoculated leaves were sampled at 24, 48 and 120 hpi. Samples were then stained with WGA (wheat germ agglutinin) for fungal growth detection. Microscopy detection of different structures of *Pst* was performed by using BX-51 microscope (Olympus, Tokyo, Japan). SV, sub-stomatal vesicle; HMC, haustorial mother cell; IH, infection hypha. H, haustoria. (**b**) Hyphal length is the average distance from the joining point of the hypha and substomatal vesicle to the peak of the hypha. DP-BSW software was used to compute the hyphal length. (**c**) The average number of haustoria mother cell per individual infection point. (**d**) The average number of haustoria per individual infection point. (**e**) The colony area per individual infection point. Data were computed from three biological replications and 50 infection sites. The significant difference between *TaAP2-15*-knockdown and control plants is indicated by asterisks, which was estimated using Student's *t*-test. *, $p < 0.05$.

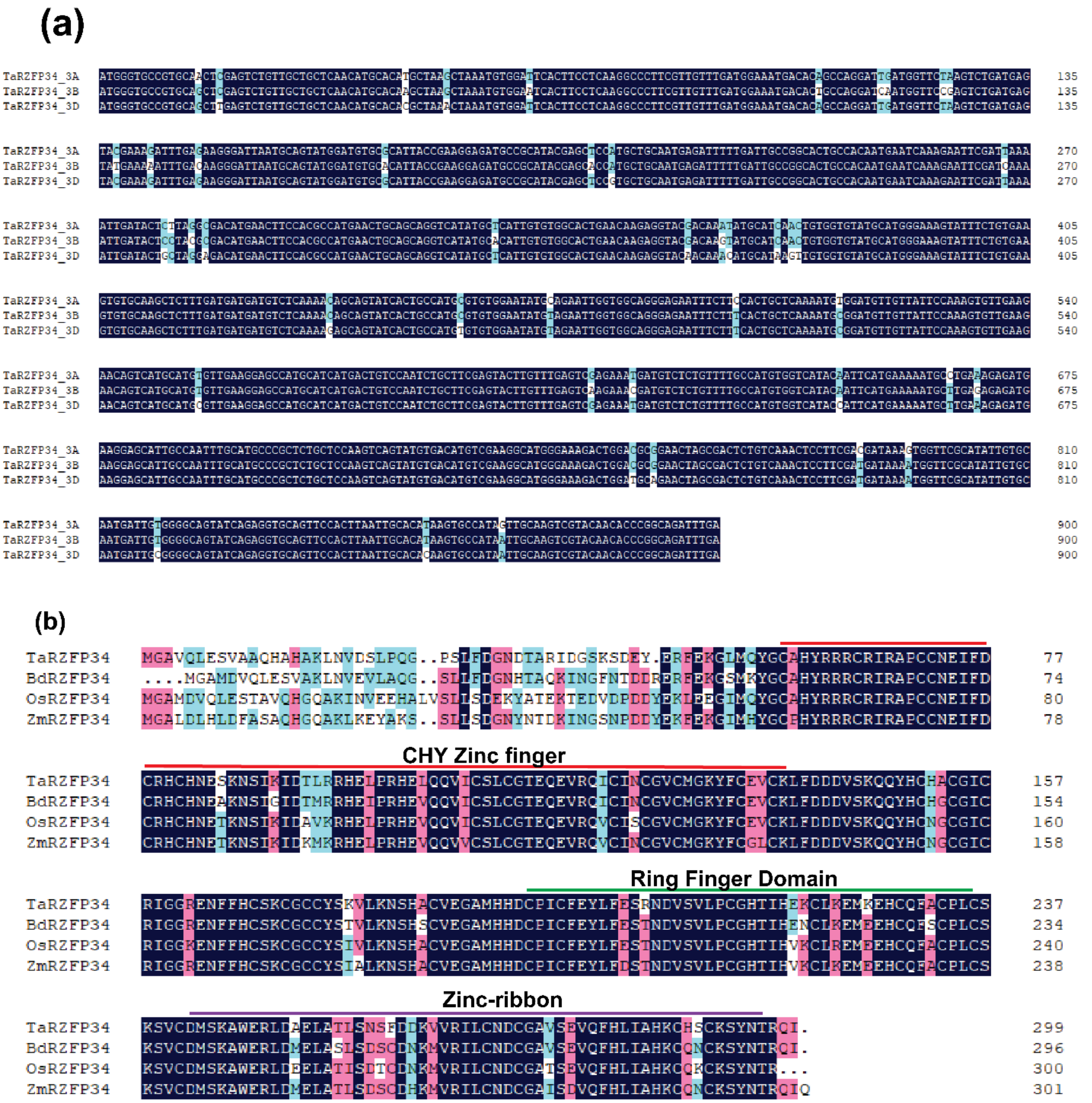

Figure S5. Multiple sequence alignment of TaRZFP34**.** **(a)** Multiple sequence alignments of the three copies of *TaRZFP34* obtained from wheat cv. Chinese Spring genome database or cloned from wheat cv. Su11. (**b**) Multi-alignment of the deduced amino acid sequences of *TaRZFP34* with its orthologs from different plant species. Comparison of amino acid sequences of *Triticum aestivum* (*TaRZFP34*), with its orthologs from *Brachypodium distachyon*, *Oryza sativum* and *Zea mays*. Conserved residues through all organisms are shown in black (100%), pink (75%-100%) and light blue (50%-75%), respectively. Sequences alignment was performed using DNAMAN8.0 software (Lynnon Biosoft, USA).

Table S1. Primers used in this study.

Function	Name	Sequence 5' to 3'
Gene amplification	TaAP2-15-S	ATGGCCAAGCAACGACGG
	TaAP2-15-AS	TCAAAGGCCCAGCTCAGC
qRT-PCR	TaAP2-15-qRT-PCR-S	CGCAGTCCAGGGAGGAGTAC
	TaAP2-15-qRT-PCR-AS	CCATTTGCCGTTGTGGTGAT
	TaPR1-S	GAGAATGCAGACGCCCAAGC
	TaPR1-AS	CTGGAGCTTGCAGTCGTTGATC
	TaPR2-S	AGGATGTTGCTTCCATGTTTGCCG
	TaCAT-S	GCCCAAGTGCTCCCACCACAACA
	TaCAT-AS	TGAGGGTGCGGGAGGGGATG
	TaEF-F	TGGTGTCATCAAGCCTGGTATGGT
	TaEF-R	ACTCATGGTGCATCTCAACGGACT
	PstEF-F	TTCGCCGTCCGTGATATGAGACAA
	PstEF-R	ATGCGTATCATGGTGGTGGAGTGA
Primer for VIGS	TaAP2-15-V1-S	TAGCTAGCTGATTAATTAAATGGCCAAGCAACGACGGA
	TaAP2-15-V1-AS	TTGCTAGCTGAGCGGCCGCTGTGCGCTGGGAGGGGC
	TaAP2-15-V2-S	TAGCTAGCTGATTAATTAAGCATCACCACAACGGCAAAT
	TaAP2-15-V2-AS	TTGCTAGCTGAGCGGCCGCCCAAACACACGCCCAATCCT
Subcellular localization	TaAP2-15-163-S	GACGATATCTCTAGAGGATCCATGGCCAAGCAACGACGG
	TaAP2-15-163-AS	GCCCTTGCTCACCATGGATCCAAGGCCCAGCTCAGCGCA
	TaAP2-15-1302-S	CATGGTAGATCTGACTAGTATGGCCAAGCAACGACGG
	TaAP2-15-1302-AS	GCCCTTGCTCACCATCCTAGGAAGGCCCAGCTCAGCGCA
Primer for Y2H	TaAP2-15-BD-S	TCAGAGGAGGACCTGCATATGATGGCCAAGCAACGACGG
	TaAP2-15-BD-AS	TCGACGGATCCCCGGGAATTCTCAAAGGCCCAGCTCAGC
	TaRZFP34-AD-S	AACATGGAGGCCAGTGAATTCATGGGTGCCGTGCAACTCG
	TaRZFP34-AD-AS	ACCACTGCTTGGGTGGAATTCAATCTGCCGGGTGTTGT
Primer for BiFC	TaAP2-15-nYFP-S	CCCAGGCCTACTAGTGGATCCATGGCCAAGCAACGACGG
	TaAP2-15-nYFP-AS	ACCCTCGAGGTCGACGGATCCAAGGCCCAGCTCAGCGCA
	TaAP2-15-cYFP-S	TGGCGCGCCACTAGTGGATCCATGGCCAAGCAACGACGG
	TaAP2-15-cYFP-S	GACAGTACTATCGATGGATCCAAGGCCCAGCTCAGCGCA
	TaRZFP34-nYFP-S	CCCAGGCCTACTAGTGGATCCATGGGTGCCGTGCAACTCG
	TaRZFP34-nYFP-AS	ACCCTCGAGGTCGACGGATCCAATCTGCCGGGTGTTGT
	TaRZFP34-cYFP-S	TGGCGCGCCACTAGTGGATCCATGGGTGCCGTGCAACTCG
	TaRZFP34-cYFP-AS	GACAGTACTATCGATGGATCCAATCTGCCGGGTGTTGT

Table S2. Candidate interacting genes screened via Y2H.

Subject name	Blast description	Gene hit	E-value	%ID
TraesCS4B02G340700.1	Methyltransferase	TraesCS4B02G340700	3.4E-129	91.9
TraesCS3A02G288900.4	Ring finger and chy zinc finger domain-containing protein 1	TraesCS3A02G288900	0.0	98.9
TraesCS7D02G323000.1	Zinc finger DHHC domain containing protein/S-acyltransferase	TraesCS7D02G323000	0.0	100.0
TraesCS5A02G164600.1	Plastid lipid-associated protein /fibrillin conserved domain	TraesCS5A02G164600	1.8E-126	96.3
TraesCS3B02G103100.1	GDSL-like Lipase/Acylhydrolase	TraesCS3B02G103100	0.12	100.0
